# A novel mutation in *STK11 *gene is associated with Peutz-Jeghers Syndrome in Indian patients

**DOI:** 10.1186/1471-2350-7-73

**Published:** 2006-09-30

**Authors:** Nikita Thakur, D Nageshwar Reddy, G Venkat Rao, P Mohankrishna, Lalji Singh, Giriraj R Chandak

**Affiliations:** 1 Genome Research Group, Centre for Cellular and Molecular Biology, Uppal Road, Hyderabad 500 007. India; 2 Asian Institute of Gastroenterology, Punjagutta, Hyderabad 500 082. India

## Abstract

**Background:**

Peutz-Jeghers syndrome (PJS) is a rare multi-organ cancer syndrome and understanding its genetic basis may help comprehend the molecular mechanism of familial cancer. A number of germ line mutations in the *STK11 *gene, encoding a serine threonine kinase have been reported in these patients. However, *STK11 *mutations do not explain all PJS cases. An earlier study reported absence of *STK11 *mutations in two Indian families and suggested another potential locus on 19q13.4 in one of them.

**Methods:**

We sequenced the promoter and the coding region including the splice-site junctions of the *STK11 *gene in 16 affected members from ten well-characterized Indian PJS families with a positive family history.

**Results:**

We did not observe any of the reported mutations in the *STK11 *gene in the index patients from these families. We identified a novel pathogenic mutation (c.790_793 delTTTG) in the *STK11 *gene in one index patient (10%) and three members of his family. The mutation resulted in a frame-shift leading to premature termination of the STK11 protein at 286^th ^codon, disruption of kinase domain and complete loss of C-terminal regulatory domain. Based on these results, we could offer predictive genetic testing, prenatal diagnosis and genetic counselling to other members of the family.

**Conclusion:**

Ours is the first study reporting the presence of *STK11 *mutation in Indian PJS patients. It also suggests that reported mutations in the *STK11 *gene are not responsible for the disease and novel mutations also do not account for many Indian PJS patients. Large-scale genomic deletions in the *STK11 *gene or another locus may be associated with the PJS phenotype in India and are worth future investigation.

## Background

Peutz-Jeghers syndrome (PJS; MIM 175200) is a rare, autosomal dominant multi-organ cancer syndrome involving primarily the gastrointestinal tract, pancreas, luminal organs, female and male reproductive organs and the lungs [1,2]. The disease is characterized by hamartomatous polyps and mucocutaneous hypermelanocytic lesions. Linkage studies have mapped the disease to 19p13.3 [3,4] and germ line mutations in the serine threonine kinase 11 (*STK11*; NM_000455.4) gene at this locus have been identified as a major cause of PJS [5-7]. STK11 is a known tumor suppressor and several studies have reported loss of the normal allele in the polyps from these patients [8-10]. Since growth of the hamartomatous polyps leads to malignancy, it is believed that inactivation of *STK11 *gene results in disruption of a fundamental growth control mechanism within somatic cells that have high proliferative capacity [11]. Penetrance of the gene is variable causing varied phenotypic manifestations among patients, which to a certain extent is correlated with the nature of the *STK11 *gene mutations [7,12]. To date, more than 140 different mutations in the *STK11 *gene have been reported and most of them result in a truncated protein but some missense or small in-frame deletions have also been reported [11]. Recent studies have reported large genomic deletions in the *STK11 *gene in about 30% of PJS patients and add to the mounting evidence for there being only one gene associated with this syndrome [13,14].

The disease has hardly been studied in the Indian population. The only study reported to date investigated two families and failed to find any mutation in the *STK11 *gene [15]. Rather, the authors identified a potential second locus (19q13.4) in one Indian family [15]. In the present study, we have recruited 10 well-characterized Indian PJS families and attempted to study the nature and importance of *STK11 *mutations in them. We have explored the 5' upstream region for putative promoter sequences and sequenced the promoter and complete coding region of the *STK11 *gene including the flanking intron-exon boundaries. We report a novel mutation in the *STK11 *gene in an Indian family and its application in prenatal diagnosis and genetic counseling for the family. Our study shows that reported mutations in the *STK11 *gene do not account for Indian PJS patients and adds to the spectrum of mutational heterogeneity associated with Peutz Jeghers syndrome. It also stresses on the role of large-scale genomic deletions in the *STK11 *gene or involvement of another PJS locus.

## Methods

### Subjects and clinical history

The classification of individuals as affected was based on the established criteria for diagnosis of PJS such as presence of mucocutaneous hypermelanocytic lesions along with more than three histopathologically proven hamartomatous polyps and a positive family history [1,2,16,17]. All ten families recruited in the study were examined by gastroenterologists (DNR and GVR) at the Asian Institute of Gastroenterology, Hyderabad and fulfilled the above-mentioned criteria. Peripheral blood samples were collected from the affected and unaffected members of these families (16 affected and 18 unaffected) after signing informed consent forms. The personal details, clinical symptoms and medical history of the patients and other family members were noted in a questionnaire at the time of collection of blood samples. In the family (Fig 1.1) that we report here, the proband was a 26-year-old man (II:2), evaluated for constipation, weakness and hyperpigmentation of lips, palms and digits. Of all members of the family, he had the earliest age of onset of symptoms at 7 yrs. He was first diagnosed to have intestinal polyps by upper GI endoscopy and colonoscopy at the age of 11 yrs. On recent investigation using capsule endoscopy, he was found to carry hundreds of polyps measuring 1 × 1.5 cm (app.) throughout the intestinal tract without any evidence of stricture. He had severe manifestations of the disease and had undergone polypectomy three times. In contrast, his elder sister (II:1) had mild disease with a relatively late age of onset at 15 years. She could not be screened for intestinal polyps at the time of this study in view of her being pregnant. The disease has been mildest in the youngest sister (aged 23 yrs) (II:3), who did not have any major complaints except for occasional instances of constipation. On colonoscopy, she was found to carry 25–30 polyps mainly in the descending and transverse colon and few in the rectum. Her small intestine could not be analyzed as she refused to give consent for upper GI endoscopy and capsule endoscopy. Their father had the first symptoms of disease at age 27, underwent his first polypectomy at 42 years and expired of severe intestinal bleeding during the course of our study. This indicates phenotypic heterogeneity, classically associated with Peutz Jeghers syndrome. Their mother (I:2) did not have history of any gastrointestinal problems. Polyps from all the patients were subjected to histopathological examination and mutation analysis for the detection of a specific mutation in the *STK11 *gene.

**Figure 1:**
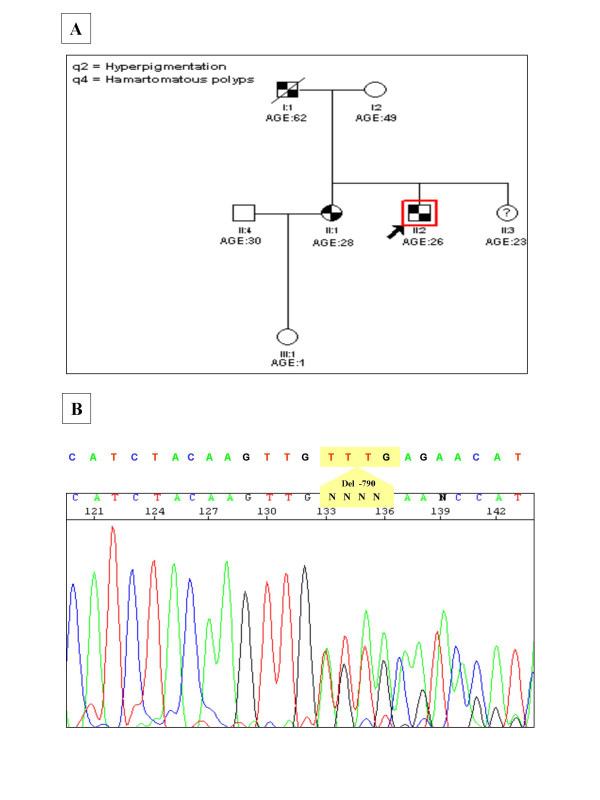
**A**, shows the pedigree chart of the Indian PJS family showing the novel 4 bp deletion (c.790_793delTTTG) in the *STK11 *gene. The proband is indicated by an arrow. The younger sister (II.3) did not show the classical symptoms of PJS and hence is represented with a "**?**". **B**, shows the electropherogram presenting a heterozygous 4 bp deletion (-TTTG) at position 790 (c.790_793delTTTG) of the *STK11 *gene as indicated by the arrow. The deletion mutation leads to a premature stop codon at 286, truncating the protein leading to partial loss of its catalytic kinase domain and complete loss of the regulatory domain.

### Characterization and analysis of the 5'UTR region of STK11

Promoter region of the *STK11 *gene is not well characterized except in a recent report [18]. We predicted a putative promoter region with the help of Transcription factor binding site (TFBS) prediction programmes, Transplorer (Biobase Biological Databases, Wolfenbuttel, Germany) and Proscan v1.7, [19] which indicated the presence of promoter elements within the regions -876 to -1126 and -1321 to -1571, position 1145787 to 1146037 and 1145341 to 1145591 respectively (NT_011255). Transplorer predicted 48 TFBSs within the region -1090 to -1472 earlier analyzed by Hearle *et al *[18]. Proscan predicted 44 TFBS in the processed sequence of 2271 base pairs upstream of the STK11 initiation signal between positions -876 to -1126 and -1322 to -1571. Promoter elements predicted by each of the program were aligned to delineate a consensus region indicating a putative promoter.

### Genetic analysis

Genomic DNA was isolated from blood and polyp tissue samples following standard protocols [20]. All nine exons with the splice site junctions and the predicted promoter region were PCR amplified with primers designed using the Genetool software. Predicted PCR products were subjected to searches of the genome using the BLAST program (21) to confirm specificity of primers (Table 1). Purified PCR products were analyzed by bi-allelic direct DNA sequencing using the Big Dye v3.1 Terminator Cycle Sequencing Ready Reaction Kit in conjunction with an ABI 3730 automated genetic analyzer (Applied Biosystems, Foster City, USA). Sequence analysis was repeated to confirm the mutation and its status in the members of the reported family. 100 normal individuals were screened for the novel mutation by sequencing. Both Centre for Cellular and Molecular Biology and Asian Institute of Gastroenterology have their Institutional Ethics Committee, which approved the study following the guidelines for research on human subjects formulated by the Indian Council of Medical Research, Ministry of Health, Government of India, New Delhi. The patients were explained various diagnostic procedures and the procedure and utility of genetic study. They were also explained about the possibility of failure to detect any mutation in the family.

**Table 1:** Primers for exon-specific sequencing of *STK11 *gene

Exon	Forward primer (5'-3')	Reverse primer (5'-3')
5' UTR	GCGTTTCTCTTTCCCCTGGTC	TGCCCTCAGCGTCCGGTCC
1	CACAAGGAAGGACCGCTCAC	CCGCTGCGACAACTGGCCTT
2 & 3	AACTCACAGCTTCTCTCTAG	AAAACTTGGGCCTTCATGTC
4 & 5	GCTGGGCCTGTGGTGTTTGG	GACGGGCCAGGCTGCACTTC
6 & 7	GCAGCCACGGGACGCCTCT	CCACCACGCCCTGCTCTAG
8	CCCTTGCACGGCCTGGTCC	TGGGACATCCTGGCCGAGT
9	TGGATACACCTGGGCCTGAC	GGGCTATGCTCACGGCTGGC

## Results and discussion

An earlier study by Mehenni *et al*., failed to detect mutations in *STK11 *gene in two multi-generational Indian Peutz-Jeghers syndrome families [15]. We sequenced the coding and the promoter regions of the *STK11 *gene in 16 patients from ten Indian PJS families. We did not find the reported mutations in any patient from these families but detected a novel mutation in one family with multiple patients in two generations. The proband in this family was found to carry a novel heterozygous 4 base pair deletion, TTTG at nucleotide position 790 in exon 6 of the *STK11 *gene (c.790_793delTTTG), which was also identified in heterozygous state in the polyps removed from his jejunum (Fig 1.2). As expected of any pathogenic *STK11 *frame-shift mutation, we did not observe this mutation in 200 normal chromosomes. Mutation analysis of other family members showed the affected father and the elder sister to be heterozygous for this mutation, both in peripheral leucocytes and the polyp tissue. The younger sister also carried the same mutation as observed in other affected family members, but she did not show the classical disease phenotype. The mutation creates a frame-shift after codon 264 resulting in premature termination of the 433 amino acid protein at 286^th ^codon. This leads to a partial loss of the kinase domain and a complete loss of the C-terminal regulatory domain (CRD). Similar 4 base pair deletions have been reported in tumor suppressor genes such as c.962_965delCTCA in BRCA1 and c.4888_4891delGTTA in APC genes associated with diseases such as breast carcinoma and familial adenomatous polyposis coli (FAP) respectively [22,23]. These mutations result in truncated proteins that are non-functional due to the loss of activity of critical functional domains.

STK11 protein mainly comprises of three major domains, the N-terminal non-catalytic domain containing the nuclear localization signal, the catalytic kinase domain and the C-terminal regulatory domain [24]. Although exact function of STK11 largely remains unknown, studies suggest its role in cell signaling and apoptosis [25]. Expression of majority of mutant STK11 proteins and assessment of their phosphorylation activity has revealed a loss of the kinase activity in *STK11 *mutants suggesting that loss of its kinase activity is probably responsible for development of the PJS phenotype [26]. We hypothesize that the deletion mutation in the reported family may lead to an altered kinase activity of the protein as a result of partial loss of the kinase domain. The resultant mutant protein is also deficient in its C-terminal domain, which is known to serve as a regulatory domain mediating dynamic interactions with several classes of proteins and promoting sub-cellular targeting apart from controlling cell polarity [27]. Mutations leading to partial or complete loss of the C-terminal domain of STK11, as observed in the present case, lead to loss of cell polarity and hamartoma formation as a result of inappropriate overgrowth of differentiated cells, which have lost their ability to regulate their polarity [27]. In the background of these observations, we propose that the deletion mutation in the *STK11 *gene in this study may contribute to polyp formation through suppression of growth arrest, apoptosis and dysregulation of AMP-activated protein kinase (AMPK) pathway leading to hyperactive mammalian target of rapamycin (mTOR) signaling [28].

Since development of tumors in most cases is a result of multiple mutations, PJS patients may have mutations in some other genes linking the *STK11 *gene to its final target for cell signaling, which may also explain phenotypic variability of the disease. Phenotypic heterogeneity and lack of genotype-phenotype correlation is a feature of Peutz Jeghers syndrome, which appears to be true for the c.790_793delTTTG mutation identified in the present study. Although, the younger sister was a carrier of the mutation, she did not have any symptoms of the disease. On colonoscopy, we found multiple intestinal polyps including three in the rectum. The polyps showed the characteristic histological features of PJS polyps, which, included irregularly dilated mucous glands with splaying of muscularis mucosa into the lamina propria and absence of dysplastic changes and also harboured the deletion mutation in heterozygous state. Taking help of this information, we advised the elder sister to undergo prenatal diagnosis by chorion villus sampling. Fortunately, the fetus was identified to be normal for the deletion mutation in the *STK11 *gene and the pregnancy was continued.

The patients recruited in this study were identified using well-established clinical diagnostic criteria for PJS (16,17). These included more than three histopathologically proven PJS polyps together with classical mucocutaneous pigmentation and a positive family history (16,17). Therefore, the possibility that these patients are affected with hamartomatous polyposis syndromes other than PJS is highly unlikely. Such syndromes include juvenile polyposis coli, Cowden syndrome or Bannayan-Ruvalcaba-Riley syndrome but none of these are characterized by typical pigmentation seen in PJS patients. The reason for absence of *STK11 *mutations in other patients is not clear, however it can be suspected that large-scale genomic deletions/insertions, undetectable by direct DNA sequencing or intronic or promoter changes in the *STK11 *gene may be responsible for the disease. Involvement of a heterogeneous minor PJS locus is another interesting possibility as suggested by Mehenni *et al*., who observed a high LOD score at 19q13.4 in one Indian family but proposed it to be due to chance only [15].

## Conclusion

We conclude that reported mutations in the *STK11 *gene are not responsible for Peutz- Jeghers syndrome in Indian patients, but novel mutations too, may not explain the disease in majority of them. Our study is the first report showing presence of a mutation in the *STK11 *gene in an Indian PJS family. However, large genomic deletions or linkage to another locus as proposed by Mehenni *et al*. are definite possibilities [15]. Extensive analysis of the *STK11 *gene using approaches such as multiple ligation dependent probe amplification may explain the disease in the remaining families. In the absence of these, genome-wide scan on these families may help to identify another PJS locus.

## Abbreviations

PJS Peutz-Jeghers syndrome

STK11 Serine threonine kinase11

CRD C-terminal regulatory domain

STRADα STE20-related adaptor

MO25 Mouse protein 25

mTOR Mammalian target of Rapamycin

AMPK AMP-activated protein kinase

TSC Tuberous sclerosis complex

TFBS Transcription factor binding site

## Competing interests

The author(s) declare that they have no competing interests.

## Authors' contributions

NT carried out molecular genetic studies including sequencing for all the families and the controls, DNR and GVR designed studies of the phenotypes, identified and diagnosed the patients, PMK did the promoter analysis, GRC conceptualized, designed the study and drafted the manuscript. All authors read and approved the final manuscript.

## Pre-publication history

The pre-publication history for this paper can be accessed here:


